# Theoretical and Experimental Analyses of the Interfacial Mechanism of Dendrimer–Doxorubicin Complexes Formation

**DOI:** 10.1021/acs.molpharmaceut.4c00941

**Published:** 2024-10-22

**Authors:** Barbara Jachimska, Magdalena Goncerz, Paweł Wolski, Callum Meldrum, Łukasz Lustyk, Tomasz Panczyk

**Affiliations:** † Jerzy Haber Institute of Catalysis and Surface Chemistry Polish Academy of Sciences, Krakow 30-239, Poland; ‡ Department of Chemical and Process Engineering, University of Strathclyde; 75 Montrose Street, Glasgow G1 1XJ, U.K.

**Keywords:** PAMAM dendrimer, doxorubicin, DDS, molecular dynamic, nanotechnology, dendrimer–drug interactions

## Abstract

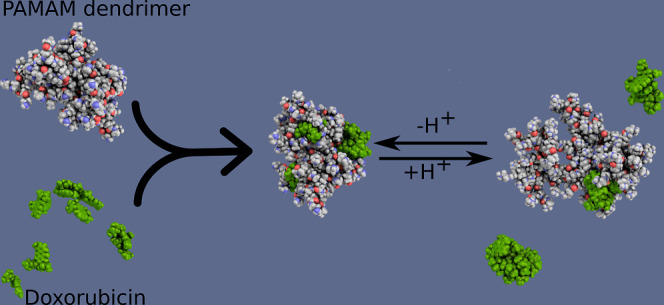

The work presents correlations between the physicochemical properties of the carrier and the active substance and optimization of the conditions for creating an active system based on PAMAM dendrimers and doxorubicin. The study monitored the influence of the ionized form of the doxorubicin molecule on the efficiency of complex formation. The deprotonated form of doxorubicin occurs under basic conditions in the pH range of 9.0–10.0. In the presence of doxorubicin, changes in the zeta potential of the complex concerning the initial system are observed. These changes result from electrostatic interactions between the drug molecules and external functional groups. Based on changes in the absorbance intensity of UV–vis spectra, the binding of the drug in the polymer structure is observed depending on the pH of the environment and the molar ratio. Optimal conditions for forming complexes occur under alkaline conditions. UV–vis, Fourier transform infrared spectroscopy, and circular dichroism spectroscopy confirmed the stability of the formed dendrimer-DOX complex. Molecular dynamics simulations were conducted to gain a deeper insight into the molecular mechanism of DOX adsorption on and within the G4.0 PAMAM dendrimers. It was observed that the protonation state of both the dendrimer and DOX significantly influences the adsorption stability. The system exhibited high stability at high pH values (∼9–10), with DOX molecules strongly adsorbed on the dendrimer surface and partially within its bulk. However, under lower pH conditions, a reduction in adsorption strength was observed, leading to the detachment of DOX clusters from the dendrimer structure.

## Introduction

An essential problem in anticancer therapies is the lack of effective drug delivery into the cell to reach cytostatic concentration while causing minimal side effects. Current treatments, such as chemotherapy or photodynamic therapy, are characterized by a long list of side effects. One way to alleviate the side effects of chemotherapy drugs is to administer them using a drug delivery system (DDS).^1^ Nanoparticle-based DDSs are promising alternatives to conventional cancer treatment methods. Understanding the relationship between the physicochemical properties of nanoparticles and their impact on the surrounding microenvironment is extremely important to achieve better functionality of these systems in the target environment. Therefore, effective nanomaterial-based systems must be appropriately designed by considering the type of nanoparticles, their surface, and their physical, chemical, and biological properties. The designed carriers’ size, shape, charge, and surface nature are the parameters that have an impact on the behavior of nanoparticles, including the type of their transport, interactions in the nanoparticle-cell system, and the way of internalization into the cell. The cell membrane creates a barrier separating the cell’s interior from the extracellular environment. This natural barrier allows for controlled transport of molecules and is an important factor influencing the effectiveness of therapies based on drug delivery to the cell’s interior.

In the past, the development of a new therapeutic agent focused mainly on its effectiveness with little or even no regard for the negative side effects that the drug can have on the patient’s body. At present, much attention is being paid to eliminating these negative side effects. The advantages of nanoparticles for drug delivery applications include controlled drug release, protection of the therapeutic payload, and improved bioavailability.^2^ Several materials have been exploited for the development of drug-loaded nanoparticles, including polymeric nanoparticles, carbon nanotubes, liposomes, polymerosomes, solid–lipid nanoparticles, gold nanoparticles, quantum dots, micelles, and dendrimers.^1−7^ Using a proper nanocarrier can significantly reduce negative side effects and increase therapeutics’ biocompatibility, specificity, shelf life, and water solubility.

Polyelectrolytes (PEs) are polymers with charged repeating monomer groups that can dissociate into a charged macroion and small counterions when dissolved in a polar solvent.^2^ They are both natural (DNA, proteins, and cellulose) or synthetic macromolecules, which present a variety of properties that make them appropriate for biomedical applications.^3−5,8−15^ Understanding the physicochemical properties of nanoparticles involved in their preparation and control of the interparticle interaction forced by adsorbing PEs is crucial from both a scientific and application-oriented point of view. One of the most critical biomedical functions of PEs is as DDS.^2,3,12,13^

Due to the increasing demand for more complex and highly specific macromolecules, the current focus is on highly branched dendritic polymers, which possess unique properties that make them ideal materials candidates for several nanoscale applications.^16–23^ Dendrimers are nanosized, nonimmunogenic, and hyperbranched polymeric systems. The dendrimer’s size, shape, molecular mass, composition, and reactivity can be precisely controlled throughout its synthesis. They have hyperbranched structures with precisely placed functional groups, allowing control of the therapeutic moiety properties encapsulated or complexed within the molecule. Among the numerous dendrimer compounds, poly(amidoamine) (PAMAM) dendrimers stand out because they were the first to be commercialized and are the most extensively characterized dendrimer family. Dendrimers can be used as nanocarriers to enhance drug solubilization, improve drug therapeutic effects, and target specific sites.

Doxorubicin (DOX) is a widely used, effective anticancer drug.^24^ However, its clinical use is limited due to high cardiotoxicity and myelosuppression.^25^ Carriers based on liposome systems containing DOX show reduced cardiotoxicity and improved specificity toward tumor cells.^26−28^ In the search for an alternative system for the delivery of doxorubicin, studies confirm the usefulness of dendrimer systems as an alternative carrier for doxorubicin. The study monitored the influence of the degree of doxorubicin ionization on the efficiency of complex formation based on the G4.0 PAMAM dendrimer. The optimal conditions for complex formation were determined by using UV–vis, Fourier transform infrared spectroscopy (FTIR), CD spectroscopy, and zeta potential measurements. The dendrimer molecule’s location preferences and interaction mechanism with the drug were analyzed using molecular dynamics (MD) simulations.

## Materials and Methods

### Materials

Fourth-generation poly(amido amine) (G4.0 PAMAM, *M* = 14.214 kDa, diagnostic grade) dendrimers in aqueous solutions (9.4% concentration) were obtained from Dendritech (Michigan; Midland, MI, USA). Dynamic light scattering (DLS) and SAXS methods analyzed the dendrimers’ size and monodispersity. The physicochemical properties of G4.0 PAMAM dendrimers are presented in Table 1. pH-controlled dendrimer solutions were prepared by diluting the starting solution in water and adding the appropriate amount of HCl or NaOH. Doxorubicin hydrochloride (DOX) was purchased from Ambeed (Arlington, Illinois, USA). A characteristic degradation of DOX in the presence of light through the photolysis effect requires the DOX and complex solutions to be completely covered, ensuring that no radiant light damages the solutions. All solutions were prepared using deionized water with ca. 1 μS/cm conductivity. Sodium chloride (NaCl), hydrochloric acid (HCl), and sodium hydroxide (NaOH) were purchased from Aldrich and Sigma. NaCl was used as the supporting electrolyte. The complex solutions were prepared by mixing the same volumes of separately prepared dendrimer and doxorubicin solutions at the same pH. pH-controlled dendrimer solutions were prepared by diluting the starting solution in water and then adding the appropriate amount of HCl or NaOH. pH-controlled doxorubicin solutions were prepared by dissolving an adequate amount of DOX in water (*c* = 184 μg/mL) and then adding the appropriate amount of HCl or NaOH. Then, the mixed solutions were put on the magnetic stirrer for 24 h of mixing (250 rpm). The dialysis process was carried out in Slide-A-Lyzer Dialysis Cassettes (MWCO = 10.0 kDa; Thermo Fisher Scientific, Waltham, MA, USA) in order to remove the unbound doxorubicin molecules. The dialysis was processed for 24 h at the magnetic stirrer in deionized water at the adjusted pH as the same pH of the complex after mixing using an appropriate amount of NaOH. The beakers in which dialysis were carried out in darkness covered with aluminum foil to eliminate the negative impact of the light. All experiments were performed at a constant temperature of 298 ± 0.1 K.

**Table 1:** Physicochemical Characterization of G4.0 PAMAM^a^

characteristic, unit	value	remarks
molecular weight [kD]	14.0	manufacturer
number of primary amine groups	64	calculated
number of tertiary amine groups	64	calculated
number of total amine groups	128	calculated
hydrodynamic radius *R*_H_ [nm]	2.45 ± 0.05 (pH = 10.2)	DLS^40^
	2.67 ± 0.05 (pH = 7.0)	
	2.79 ± 0.05 (pH = 4.3)	
gyration radius *R*_g_ [nm]	1.87 ± 0.02 (pH = 10.2)	SAXS^40^
	2.11 ± 0.02 (pH = 7.0)	
	2.17 ± 0.02 (pH = 4.3)	
gyration radius *R*_g_ [nm]	1.47 ± 0.01 (no protonation)	molecular dynamics^40^
	1.46 ± 0.01 (10% protonation)	
	1.54 ± 0.02 (20% protonation)	
p*K*_a_	8.0	potentiometric titration^41^
i.e.p. isoelectric point	9.9	electrophoretic mobility^39^
max effective charge *N*_c_	11.5	calculated from electrophoretic mobility

a Note: the effective degree of ionization of a molecule is given by α = *N*_c_/*N*_m_, where *N*_m_ is the nominal number of charges per molecule and *N*_c_ is the average number of free charges (of positive sign) per PAMAM dendrimer molecule.^37^

### DLS and Zeta Potential

DLS was used to determine the size of the G4.0 PAMAM molecule and the size and stability of the PAMAM-DOX complexes. Measurements were performed on a Zetasizer Nano ZS device (Malvern Instrument, UK). The device is equipped with a HeNe laser, which is linearly polarized, performing measurements at a wavelength of 632.8 nm and angles of 173° and 130° for size and zeta potential measurements, respectively. The average hydrodynamic diameter and polydispersity index (PDI) were determined from the diffusion coefficient of particles subjected to Brownian motion. Measurements were performed 10 times. The laser Doppler velocimetry technique was used to evaluate the zeta potential by measuring the electrophoretic mobility of the samples and employing the Helmholtz–Smoluchowski equations. Measurements were performed 5 times.

### UV–Vis Spectroscopy

UV–vis spectra for complexes were obtained by the Thermo Scientific Evolution 201 UV–vis spectrophotometer in the wavelength range of 190–800 nm with a 2 nm slit width and a 1 cm path length at intervals of 1 nm with the solvent as a baseline. UV–vis spectroscopy was used to control the concentration and tautomeric form of doxorubicin hydrochloride in water and to determine the efficiency of the G4.0-DOX complex formation.

### Circular Dichroism

Circular dichroism was performed on a JASCO J-1500 circular dichroism spectrophotometer with a 150 W Xe lamp. The data were recorded between 185 and 290 nm wavelength with a 0.025 nm data pitch, 50 nm/min scanning speed, and 1 nm bandwidth. The samples were analyzed in a 1 mm path-length quartz rectangular cell using 5 repetitions. Circular dichroism was used to analyze changes in the structure of doxorubicin hydrochloride in water depending on the concentration and effectiveness of complex formation.

### Quartz Crystal Microbalance

Quartz crystal microbalance (QCM-D) measurement was done using a Q-sense E4 instrument (Västra Frölunda, Sweden). QCM-D measurements simultaneously measure changes in frequency (Δ*f*) and energy dissipation (Δ*D*) during adsorption onto the sensor surface. The decrease in the crystal oscillation frequency indicates that an adsorption process occurred on the sensor surface. The Sauerbrey equation was applied in the field of rigid layers adsorbed on the sensor surface for overtone *n* = 7 using QTools software. In the case of viscoelastic layers, the adsorbed mass was calculated using the Voigt model.^29^ The QSense DFind, Biolin Scientific, Espoo, Finland for 3-11 frequency overtones software was used in this case. The second parameter monitored during the QCM-D experiment is energy dissipation, which is related to the viscoelastic properties of the formed layer. All experiments used QCM-D (Q-sense) sensors with a thin layer of gold on the surface.

### Fourier Transform Infrared Spectroscopy

FTIR measurements were performed using a Nicolet iS50, Thermo Fisher Scientific, MA/USA FTIR spectrometer with a (SR) SMART SAGA attachment. FTIR spectra were recorded in the wavenumber range from 700 to 4000 cm^–1^. Sample spectra were obtained by averaging 512 scans with a spectral resolution of 4 cm^–1^. Before each measurement, the spectrum of the initial surface was recorded and automatically subtracted from the sample spectrum. Omnic software (Thermo Fisher Scientific, MA/USA) was used to analyze the spectra.

### Molecular Dynamics Simulations

The initial structures of G4 PAMAM dendrimers, at different protonation levels, were constructed using a self-developed dendrimer topology builder based on the approach proposed by Maingi et al.^30^ The energetics parameters for internal, intermediate, and terminal branches of PAMAM dendrimers were obtained from the GAFF force field.^31^ To adjust the charge distribution within both types of dendrimers, each dendrimer building block was optimized using the HF/6-31G(d) level of theory, and charge distribution was obtained from the restrained electrostatic potential (RESP) method using the R.E.D server.^32^ The same strategy was utilized to obtain the parameters and atomic partial charges of two types of DOX molecules.

To generate the relaxed structures of both types of PAMAM molecules, the initial configurations of dendrimers were placed in a box of explicit TIP3P water and then subjected to minimization, equilibration, and production runs of standard MD simulations using GROMACS^33^ software. Next, each equilibrium structure of the dendrimer was again placed in the water box, surrounded by 10 DOX molecules that were randomly distributed in the box, and the MD simulations were carried out with the following protocol: (1) 1500 steepest descent minimization steps; (2) 1 ns *NVT* simulation at 300 K; (3) 1 ns of *NPT* simulation at 300 K and 1 bar; (4) 10 ns of unrestrained *NPT* simulation; and finally (5) 50 ns for G4N-DOX(−) and 100 ns for G4P-DOX(+) of unrestrained *NPT* dynamics at 300 K and 1 bar from which simulation data were collected. For all simulations, a V-rescale thermostat and a Parrinello–Rahman barostat were used. The LINCS algorithm was employed to constrain all bonds involving hydrogen. Long-range electrostatics were treated by using the Particle-Mesh Ewald approach. The Newtonian equations of motion were integrated using the leapfrog scheme with a time step of 2 fs. In both cases, periodic boundary conditions were adopted. The convergence of the systems was monitored by tracking the potential energy and RMSD values (see Figure S1 in the Supporting Information).

To estimate the adsorption capacity of DOX molecules to dendrimers at different pH levels, we calculated the binding free energy Δ*G* using the Molecular Mechanics/Poisson–Boltzmann Surface Area (MM-PBSA) method,^34^ which is implemented in the gmx_mmpbsa script.^35^

## Results and Discussion

### Physicochemical Characterization of the System

Dendrimers are often used in nanomedicine (cancer therapy or diagnostic agent) and pharmacy (carriers of drugs, genes, or bioactive substances).^16−20^ Dendrimers have unique properties arising from their macromolecular structure. Dendrimers undergo irreversible swelling, which is directly related to the degree of protonation of the dendrimer’s functional groups.^36^ Dendrimers are characterized by a high degree of hydration, which may be an important advantage when using them in biological systems.^37^ The key physicochemical parameters of G4.0 PAMAM dendrimers are given in Table 1. The stability of dendrimer solutions was monitored using the DLS method. Their hydrodynamic radius was determined based on measurements of the diffusion coefficient of dendrimer solutions. DLS measurements highlighted a strong dependence on dendrimer particle size relative to solution pH. The hydrodynamic radius of G4.0 PAMAM molecules in the pH range 10.0–4.0 in aqueous solution ranges from 2.45 to 2.79 ± 0.05 nm.^38,39^ In the range of extreme pH values > 10, lower values of the hydrodynamic radius are observed, associated with a decrease in the protonation of functional groups in the dendrimer structure. Similar values for the G4.0 molecule were obtained using the SAXS method.^38^ In this case, the gyration radius varies in the 2.1–1.87 ± 0.02 nm range as the pH goes from acidic to basic. Our previous theoretical study showed that at basic pH, even a slight increase in the number of protonated amines (up to 20%) can affect the size of dendrimers^40^ (see Table 1).

PAMAM dendrimer is a weak polyelectrolyte whose charge can be adjusted depending on the pH or ionic strength of the solution. Electrophoretic mobility (μ_e_) measurements allow us to determine the effective degree of ionization of the dendrimer molecule and the isoelectric point of the tested system.^36−38^ Changes in electrophoretic mobility, zeta potential, and effective charge of the fourth generation PAMAM dendrimer molecule are summarized in Table 1. The effective charge and its dependence on pH and ionic strength are crucial to determining the optimal conditions for forming dendrimer-based complexes. PAMAM G4.0 dendrimers contain primary (*N*_primary_ = 64) and tertiary amino groups (*N*_tetirary_ = 64) in their structure. These groups determine the protonation mechanism of the PAMAM dendrimer molecule. At low pH, all primary and tertiary amines are protonated (pH < 4). Primary amines in the molecule’s structure are protonated at intermediate or neutral pH. At high pH values, no protonation is observed. This is confirmed by the location of the isoelectric point of the dendrimer at pH = 10.0 (Figure 2A). Dendrimer molecules contain two types of amino groups simultaneously located in different chemical environments. As a result, primary groups are much more basic than tertiary groups. Due to the weak interaction between both types of functional groups, two kinds of groups are protonated almost independently of each other. All primary amines are protonated in the basic region. However, both types of functional groups are protonated under more acidic conditions. On this basis, it is known that at high pH, the outer layer of the dendrimer molecule is protonated, while the dendrimer core is protonated only at lower pH. The pH value at which 50% of the functional groups are ionized (p*K*_a_) for the dendrimer occurs at pH = 8.0.^41^

**Figure 1:**
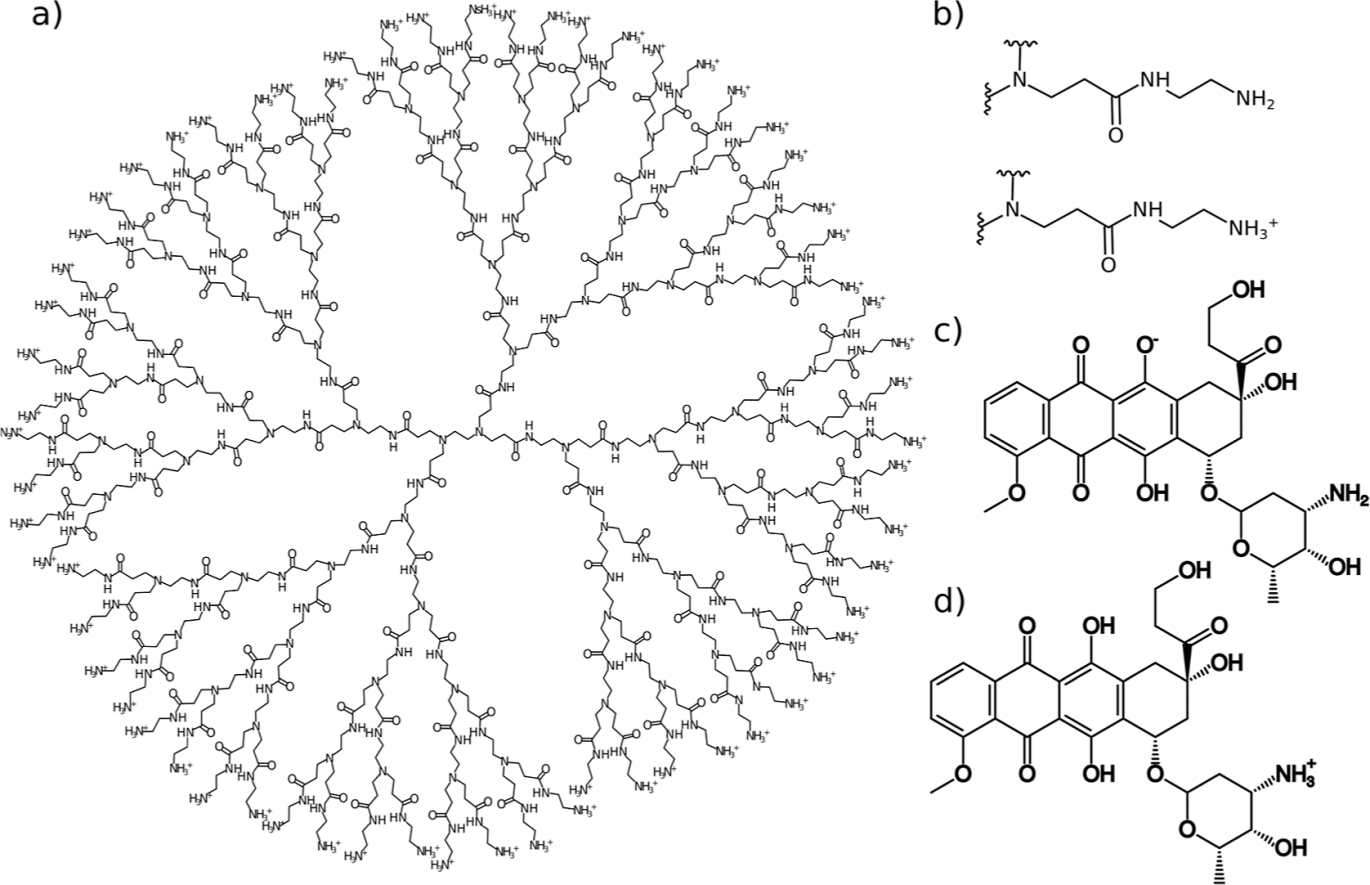
(A) Structure of a fourth-generation PAMAM dendrimer (G4.0 PAMAM). (B) Structural representation of a terminal branch of a PAMAM dendrimer in nonprotonated and protonated forms. (C) Structural representation of a doxorubicin molecule in its ionized form, DOX–. (D) Structural representation of a doxorubicin molecule in its protonated form, DOX+.

**Figure 2:**
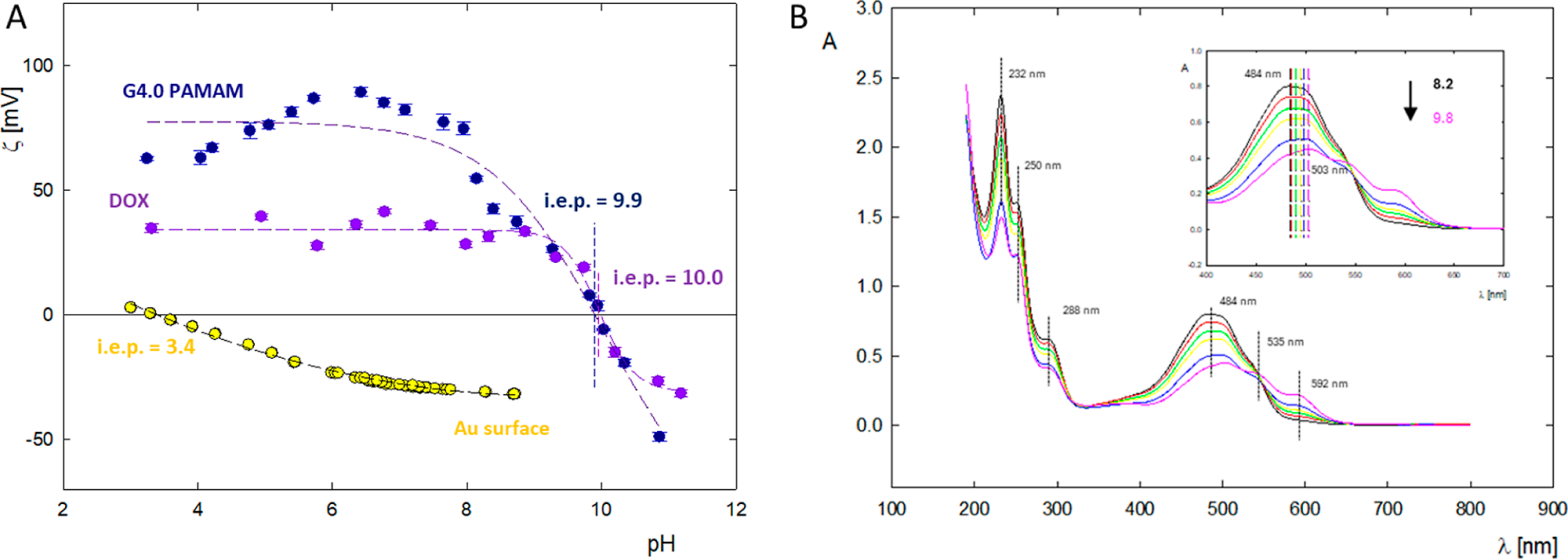
(A) Zeta potential of G4.0 PAMAM, doxorubicin, and gold surface dependent on pH. (B) UV–vis spectra of doxorubicin (*c* = 50 ppm) in the pH range 8.2–9.8.

To reduce therapeutic agents’ side effects, their targeting efficiency must be improved. One effective strategy is immobilizing the ligands on the surface or inside the support. The ligand tested in this study is doxorubicin, an antibiotic from the anthracycline family with cytostatic activity. Doxorubicin is amphiphilic and can exist in various forms in an aqueous solution, protonated and deprotonated, depending on the pH of the environment.

The critical structural feature common to anthracyclines is the tetracyclic anthraquinone ring system, which constitutes the hydrophobic aglycone core of these molecules and is the chromophore responsible for their characteristic red color (λ_max_ = 480 nm). The molecule has asymmetric carbon atoms in aglycone and daunosamine. The unsaturated ring in the aglycone can assume various conformations, and the glycosidic bond assumes a specific angle between the sugar group and the aglycone. Doxorubicin contains three dissociable protons, one in the ammonium group of daunosamine and two in the phenolic hydroxyl groups of the 1,4-dihydroxyanthraquinone moiety. The p*K*_a_ value of daunosamine occurs at pH = 8.2. The p*K*_a_ of two phenols is shifted toward higher values of pH = 9.5, with the hydroxyl group located closer to the sugar group in the structure being more basic.^43,44^

The anthraquinone ring containing the amino sugar daunosamine also has a geminal tertiary hydroxyl group and a ketone side chain. The substituents described above play a crucial role in the context of the biological activity of anthracyclines. Fiallo et al.^45^ presents an analysis of the spectra of 17 anthracycline derivatives and determines individual electronic transitions. The bands corresponding to the π → π* transition, polarized along the short axis of the anthraquinone (∼290 nm), do not depend on the ionization state of the phenolic hydroxyl groups, while the position of the band corresponding to the π → π* transition (∼480 nm), polarized along the long axis of anthraquinone, depends strongly on them. The *n* → π* transitions occur at ∼320–350 nm depending on the C=O groups located in the quinone.

Based on measurements of the zeta potential of the aggregated form of doxorubicin, the charge of the drug, depending on environmental conditions, was determined. In a wide pH range of 4.0–9.0, DOX has a high positive zeta potential of ζ = 40 ± 5 mV. Above pH 9.0, a gradual decrease in the molecule’s charge is visible. In an aqueous solution, the isoelectric point for DOX occurs at pH 9.9. For pH > 10.0, the drug molecule has a negative zeta potential ζ = −25 ± 1 mV (Figure 2A).^42^ Changes in the charge of the doxorubicin molecule result directly from the degree of ionization of individual functional groups present in the drug molecule. The positive charge is attributed to the protonation of the amino group of daunosamine in an acidic environment. However, as the pH increases, the share of deprotonated forms of phenolic groups will cause a change in the charge of the molecule.

Figure 2A shows the pH dependence of the surface zeta potential for the gold sensors used in the QCM-D adsorption experiments. The charge density of the gold surface is sensitive to the pH of the solution and approaches the zeta potential of −32.5 ± 1 mV at pH 5.5.^46^ In this condition, the charge density is equal to −0.016 e/nm^2^. The gold sensor is highly charged at high pH and weakly charged at low pH. The isoelectric point for the gold surface is near pH 2.8.^47^ For comparison, the charge density of the dendrimer molecules changed from 0.081 e/nm^2^ at pH 4.0 to −0.019 e/nm^2^ at pH 10.0. It should be noted that the dendrimer particles and the gold surface are oppositely charged. Therefore, the dendrimer particles can be adsorbed very efficiently on the gold surface.

The fluctuations in absorbance value and shift in the maximum across the pH range 8.2–9.8 are shown in Figure 2B. The absorption spectra of the drug depict three bands in the visible region (592, 535, and 484 nm) and three in the ultraviolet region (288, 250, and 232 nm).^27^ Three visible bands of the region of conjugated anthracycline rings in the ultraviolet region are characteristic; one at 288 nm indicates the aromatic ring, and the remaining two, i.e., 250 and 232 nm, refer to the sugar residue of daunosamine. With an increase in pH of the doxorubicin solution, there is a decrease in the absorption intensity of the spectrum and a simultaneous bathochromic shift in the location of the spectral maxima in the range of 480–600 nm. In an alkaline environment, the most significant changes are visible in terms of spectral intensity and the maximum position, which shifts toward longer wavelengths from 484 to 503 nm. Due to the change in the position and value of the spectral maximum, which depends on the degree of ionization of the DOX molecule, extinction constants were determined for individual pH values, which were used to determine the drug concentration in the complexes before and after dialysis. The extinction constants for the doxorubicin solution are ε = 9610 M^–1^ cm^–1^ for pH 7.5.^38^

### G4.0 PAMAM-DOX Complex Formation

The nature of the surface groups of dendrimers determines the interactions of these carriers with the ligand molecules. Considering that G4.0PAMAM and DOX dendrimers in a similar pH range have a very high positive charge, it is expected that the effective formation of the PAMAM-DOX complex will take place under conditions where both components have a low charge.

**Figure 3:**
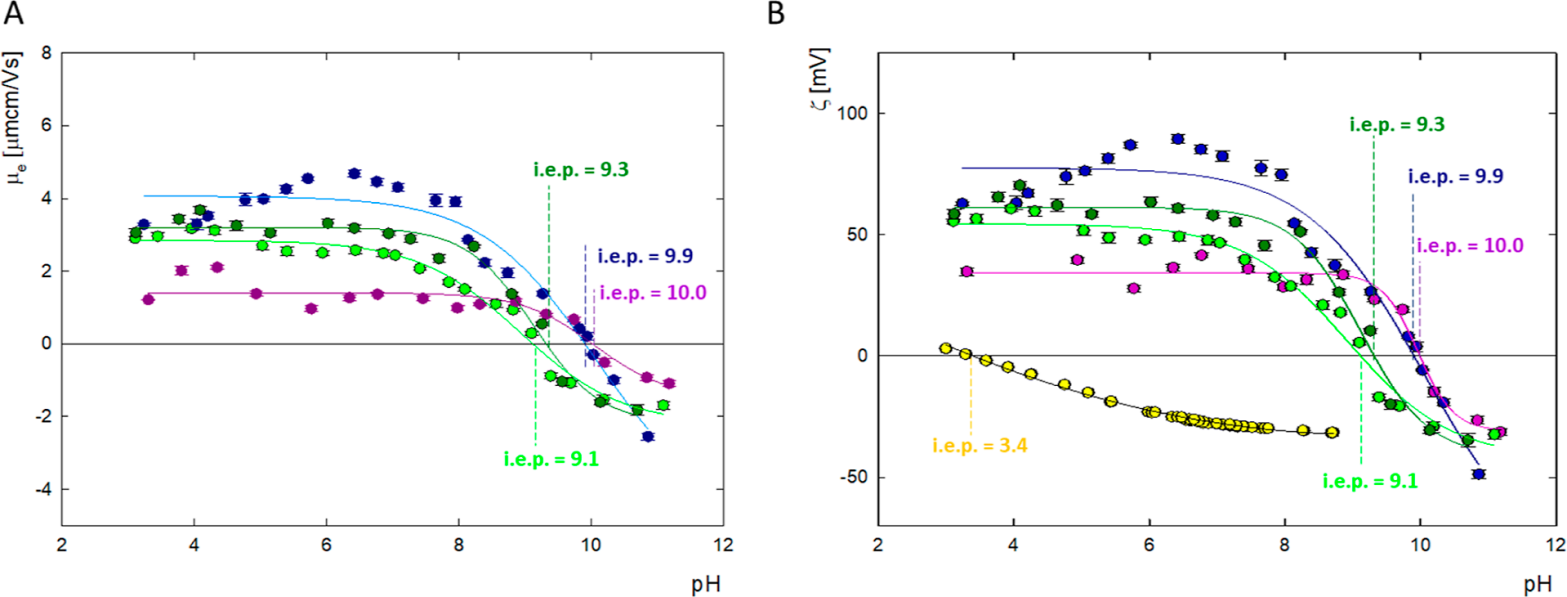
Physicochemical characterization of G4.0 PAMAM dendrimer, doxorubicin, and complexes before and after dialysis for molar ratio G4.0 PAMAM/DOX = 1:9. (A) Change in the mobility (μ_e_); (B) zeta potential (ζ) (G4.0 PAMAM—dark blue line, G4.0PAMAM/DOX before dialysis—light green line, G4.0PAMAM/DOX before dialysis—dark green line, doxorubicin—purple line, and gold surface—yellow line).

Based on the changes in the zeta potential of dendrimers and doxorubicin, complexes were formed in the pH range 9.0–10.0 (Figure 3). G4.0 PAMAM complexes with doxorubicin were formed in an aqueous solution at a constant dendrimer concentration of 0.25 mg/mL (17.6 μM). The molar ratio of carrier to the drug was tested in the range of 1:6 to 1:24, and the initial pH of the complexes was adjusted appropriately at pH = 9.0, 9.5, or 10.0. All complexes were mixed for 24 h and then subjected to dialysis. The formation of the complex was monitored in many ways by using UV–vis, CD, FTIR spectroscopy, and zeta potential measurements pre- and postdialysis. Because some of the drug molecules may not be permanently bound to the carrier, the complex formed was monitored immediately after formation and after the dialysis process. Changes in zeta potential before and after dialysis have a similar course over the entire pH range. The zeta potential of the complex is lower than the zeta potential of the dendrimers and higher than the zeta potential of the drug. After dialysis, a slight increase in zeta potential is observed compared to the complexes before dialysis. In the pH range 4.0–6.0, DOX is in the protonated form and if it is not permanently bound to the carrier, it is electrostatically repelled from the dendrimer molecule. Changes in the value of the zeta potential in relation to the initial system indicate that some of the drug molecules have been immobilized on the surface of the dendrimer structure. Adding the drug to the system shifts the isoelectric point from pH = 9.9 toward lower values of pH = 9.1 before dialysis and pH = 9.3 after dialysis, respectively. The change in i.e.p results from the specific adsorption of drug molecules onto the carrier surface.

**Figure 4:**
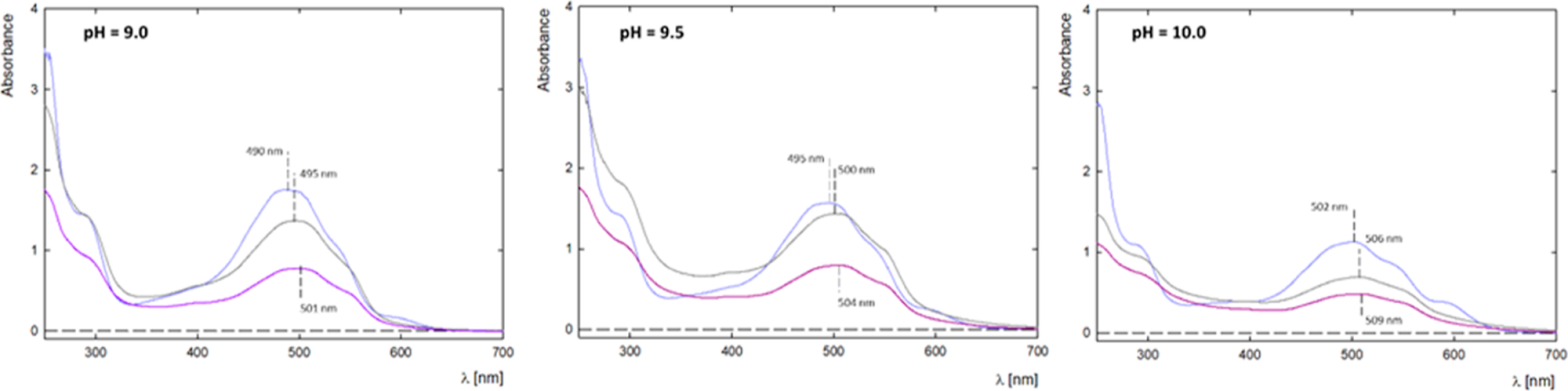
UV–vis spectra G4.0 PAMAM/DOX complexes before and after dialysis for molar ratio 1:9 at (A) pH 9.0; (B) pH 9.5 and (C) pH 10.0 (light purple line-G4.0PAMAM/DOX before mixing, gray line-G4.0PAMAM/DOX before dialysis and purple line-G4.0PAMAM/DOX-after dialysis).

Complex formation was monitored by using UV–vis spectroscopy. The efficiency of complex formation at pH 9.0, 9.5, and 10.0 is presented in Figure 4. After complex formation, slight changes in the peak intensity and a slight shift in the maximum position from 490 to 501 nm are visible. After 24 h of mixing, there is a significant decrease in the signal, especially in the case of pH 10.0, the smallest decrease in intensity was obtained for pH 9.5. After dialysis, a further decrease in the signal is observed for all complexes. The highest absorbance intensity for the complex was obtained at pH 9.5. It should be noted that in the pH range 9.0–9.5, there are large changes in the ionized form in which the drug occurs. The stoichiometry of the formed complexes was determined based on UV–vis spectra. For complexes formed in a molar ratio of G4.0 PAMAM/DOX 1:9, after the dialysis process, obtain a ratio of 1:4.2 at pH = 9.0, ratio of 1:4.2 at pH = 9.5, and 1:2.6 at pH = 10.0, respectively. The encapsulation efficiency (EE) equals 28–47% and loading content (LC) 10–17%. The EE is the ratio of the mass of the drug incorporated into the carrier to the initial mass of the drug and LC values, i.e., the ratio of the mass of the drug incorporated into the carrier to the mass of the carrier.

A functional quantifiable value for studying aggregation is the binding coefficient, which is used to determine the extent to which a chemical compound binds receptor molecules. The binding coefficient can be determined by using a nonlinear regression method to solve the Hill–Langmuir equation,^48,49^ which defines the cooperativity between a receptor molecule (dendrimer) and binding molecules (DOX).
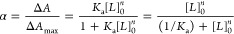
1where Δ*A*_max_ is the maximum absorbance deviation, Δ*A* (=*A*_obs_ – *A*_0_) is the change in absorbance, [*L*]_0_ is the guest concentration (DOX), *n* is the Hill coefficient, and *K*_a_ is the association binding constant.

The absorbance peak, as taken from UV–vis, was used in Sigma Plot V10 software, and by using its sophisticated regression tools, a Hill–Langmuir nonlinear fit was used to solve eq 1. The values of the binding constant are equal. *K*_a_ = (2.39 ± 0.43) × 10^2^ M^–1^ for pH 9.0 and *K*_a_ = (2.73 ± 0.16) × 10^2^ M^–1^ for pH = 9.5.^38^ A study of the G4.0 PAMAM-DOX complex system under physiological conditions using fluorescence spectroscopy determined a binding constant of *K*_a_ = 1.6 × 10^6^ M^–1^.^42^ The lower the binding constant, the higher the affinity between a receptor and conjugate since the binding constant alludes to the required conjugate concentration for effective binding to the receptor. The results show that the binding constant is heavily affected by the pH of the solution since there are several orders of magnitude differences between it at initial pH = 5.7, 9.0, and 9.5. This indicates a substantial increase in the drug affinity to the carrier with increased pH. Determination of the value of Hill’s coefficient (*n*) provides information on the cooperativity of the binding process of doxorubicin to the G4.0 PAMAM structure under different conditions. When Hill’s coefficient equals 1, it suggests independent binding and lack of cooperativity. Values for which *n* ≠ 1 indicate multiple ligand binding corresponding to negative (*n* < 1) or positive (*n* > 1) cooperativity. In the case studied, the results suggest noncooperativity of doxorubicin binding by the dendrimer carrier for all pH values.

**Figure 5:**
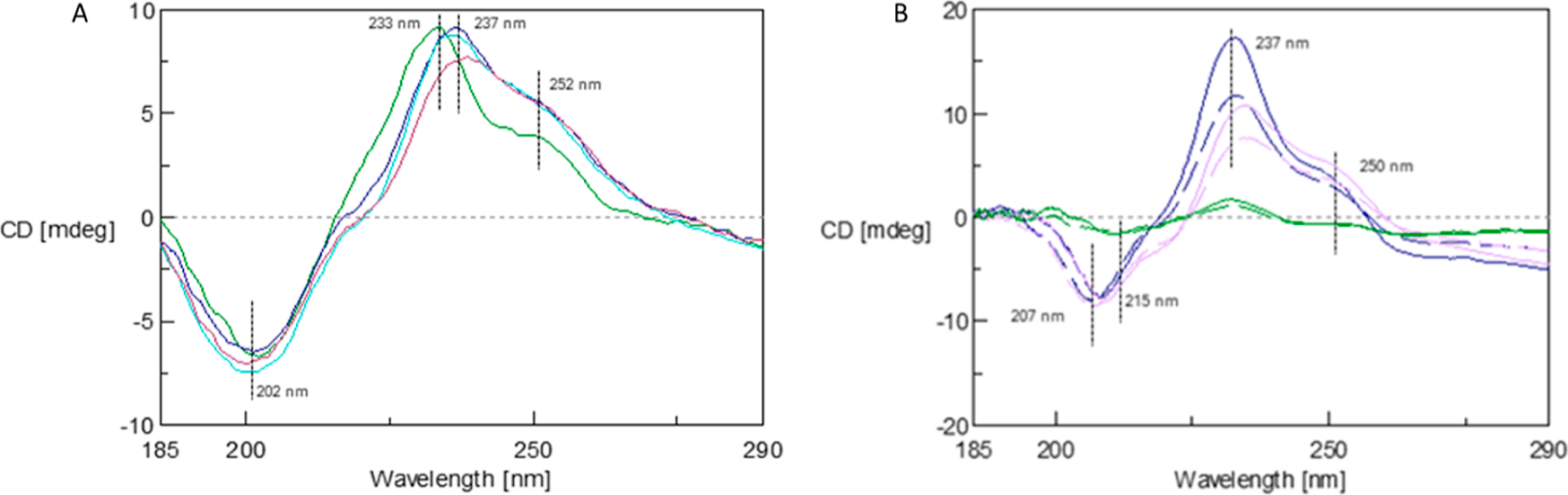
CD spectra for (A) DOX solution in water at a concentration of 0.1 mg/mL in pH 5.7 (green line), 9.0 (violet line), 9.5 (blue line), and 10.0 (dark red line) and (B) G4.0 PAMAM-DOX complexes 1:9 depending on the pH after 24 h of mixing (blue line—pH = 9.0, purple line—pH = 9.5, and green line—pH = 10.0; solid lines—complex before dialysis, dashed lines—complex after dialysis).

The efficiency of complex formation was monitored by using CD spectroscopy. The CD spectrum of doxorubicin in the 185–290 nm range has one minimum at 202 nm and two maxima at 233 and 250 nm. As the pH increases, a shift of the maximum of the 233 nm spectrum toward 237 nm is observed and its intensity decreases. The maximum at 250 nm does not change its position; only its intensity increases. Figure 5B shows the spectra of the complexes for the 1:9 ratio formed at pH 9.0, 9.5, and 10.0 before and after dialysis. As a result of doxorubicin interaction with the dendrimer molecule, the positions of both the minimum and maximum of the spectrum change. Particularly large changes are visible in the spectra of complexes formed at pH 9.5, for which the maximum at 237 nm decreases but the maximum at 250 nm increases significantly.

The size of the complexes was controlled by using the DLS method. DLS measurements confirmed the tendency of the system to form aggregates. As the pH increases, an increase in the size of the aggregates formed is observed. For pH 9.0, the size of the aggregates is 581 nm before dialysis and 190 nm after dialysis. PDI values indicate the polydisperse nature of the system. Above pH = 9.0, aggregation increases strongly to values above 1000 nm, which the zeta potential values of both dendrimers and doxorubicin may favor. Additionally, doxorubicin has a natural tendency to form aggregated forms. The aggregation process of doxorubicin increases intensively with increasing drug concentration and environmental pH.^50^

**Figure 6:**
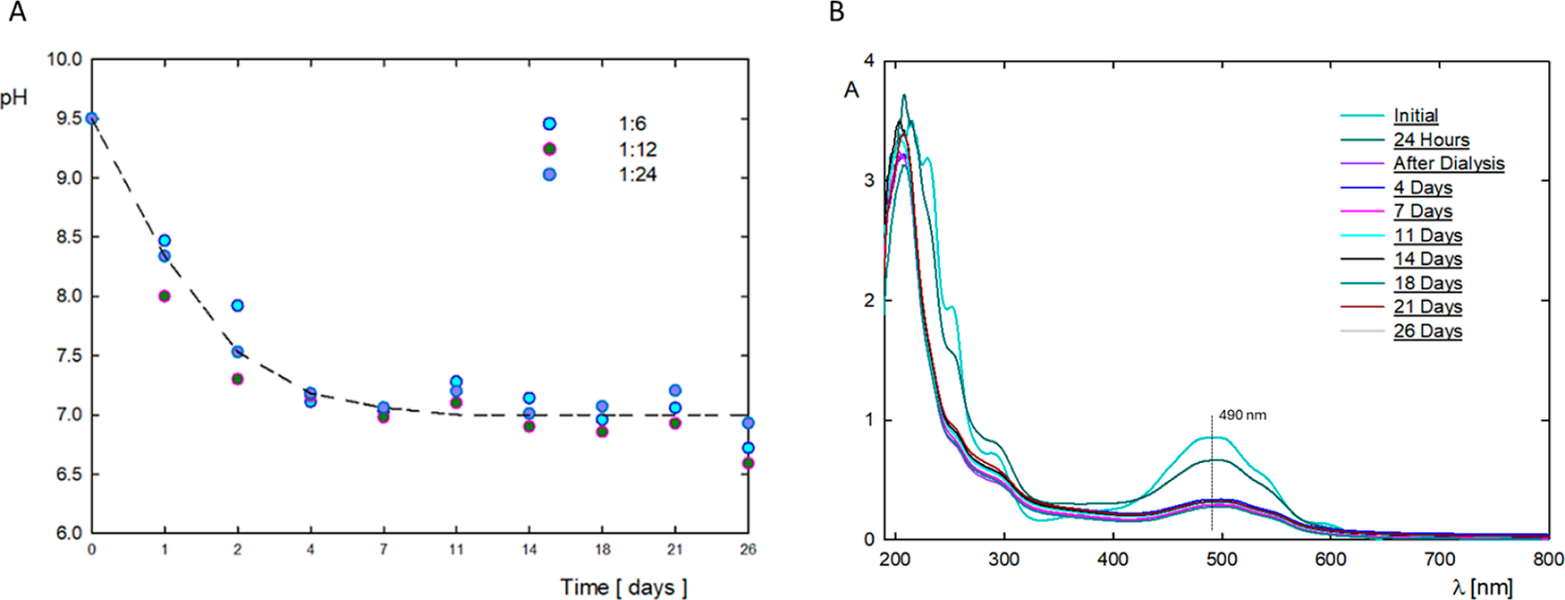
(A) Changes over time in the pH of solutions of G4.0/DOX complexes formed at pH 9.5 for molar ratios of 1:6, 1:12, and 1:24. (B) Stability of G4.0/DOX complexes formed at pH 9.5 in a molar ratio of 1:12 monitored by changes in the UV–vis spectrum.

In the case of drug carriers, an important parameter of the system is the stability of the physicochemical properties over time. The physicochemical properties of the G4.0 PAMAM/DOX system with molar ratios of 1:6, 1:12, and 1:24 were tested for 26 days. Figure 6 shows the changes in the pH of the system and the intensity of the UV–vis spectrum depending on the incubation time. In the tested time range, the same trend in changes in the pH of the system was obtained for all three molar ratios of the components. The greatest drop in the pH of the system is observed in the first 4 days after the formation of the complex. The pH level after 4 days from creation remained constant for up to 26 days. As for changes in the maximum absorbance intensity at a wavelength of 490 nm, the greatest changes are observed after dialysis during which unbound drug molecules are removed from the system. After dialysis for 26 days, no significant changes in the UV–vis spectra were observed for all tested complexes, regardless of the initial molar ratio of the components.

To demonstrate changes like the carrier properties after its functionalization, wettability tests of the system were carried out. Contact angle measurements were carried out on the hydrophobic gold surface, with a contact angle of 75° ± 4. The adsorption surface was selected due to the contact angle in the range corresponding to the conditions of adhesion to the cell surface. Modification of the gold surface with a G4.0 PAMAM dendrimer resulted in a reduction in contact angle to 61° ± 7. The doxorubicin layer is more hydrophilic than dendrimers adsorbed on the surface. The contact angle for the DOX layer is 54° ± 1. The layer of complexes of dendrimers with the drug has a similar contact angle to the drug layer, respectively, 57° ± 1 at pH = 9.0 and 56° ± 1 at pH 9.5.

**Figure 7:**
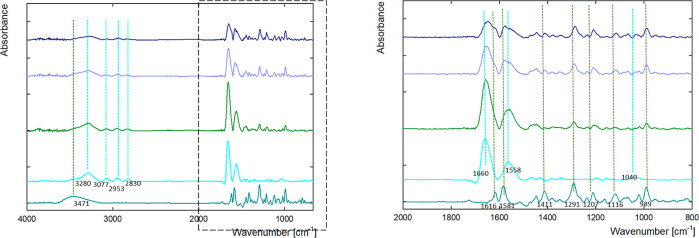
FTIR spectra of G4.0-DOX ratio 1:9 (17.6 μM: 158.3 μM) complexes after dialysis (green line pH = 9.0, light purple line pH = 9.5, and dark blue line pH = 10.0) compared to free DOX (dark cyan line) and G4.0 PAMAM (light blue line) at pH 9.5 in ranges (A) 665–4000 and (B) 800–2000 cm^–1^.

Additionally, the doxorubicin dendrimer interaction was determined using infrared spectroscopy. The FTIR spectra of G4.0 PAMAM, DOX, and G4.0 PAMAM-DOX complexes after dialysis are shown in Figure 7. Dutta et al. characterized major peaks in the IR spectrum of fourth-generation PAMAM dendrimers in phosphate buffer saline: 3473.9 cm^–1^ (N–H asymmetric stretch primary amine), 3440 cm^–1^ (N–H symmetric stretch primary amine), 2975.9 cm^–1^ (C–H stretch), 1731.5 and 1692.5 cm^–1^ (C=O stretch amide I band), 1599.9 cm^–1^ (N–H in-plane bending amide II band), 1285.5 cm^–1^ (C–N stretch amines), 630.1 cm^–1^ (OCN deformation amide IV band), and 1109.6 and 1052.7 cm^–1^ (C–C bend).^51^Figure 7 shows the FTIR of a G4.0 PAMAM dendrimer solution with a concentration of 250 ppm in water. It can be seen in the samples that the peaks at 3077, 2953, and 2830 cm^–1^ were typical C–H stretch vibrations. A broad absorption band at 3380 cm^–1^ was attributed to the stretching vibration of –NH_2_. An increase in intensity was observed for amide I at 1660 cm^–1^ and amide II at 1548 cm^–1^. The spectra of doxorubicin have a broad band in the 3471 cm^–1^ range (N–H stretch) and two peaks in the amide range (amide I at 1616 cm^–1^ and amide II at 1581 cm^–1^).

The dendrimer antisymmetric and symmetric CH_2_ stretching vibrations in the 3000–2800 cm^–1^ IR spectra region were used to examine the hydrophobic contact in the dendrimer-DOX complexes. The CH_2_ band for free G4.0 PAMAM located at 2953 and 2830 cm^–1^ is visible in the dendrimer-DOX complexes. The doxorubicin peaks in the amide I and amide II ranges have shifted.

### Effect of Dendrimer-Based Interlayer for Doxorubicin Immobilization

The QCM-D method was used to monitor the interaction of doxorubicin with PAMAM G4.0 dendrimers under dynamic conditions. QCM-D is a technique used to measure the adsorption of macromolecules, proteins, and nanoparticles at a liquid–solid interface. The operation is based on fluctuations in the resonance frequency of a quartz crystal as a result of the increase in mass during adsorption onto the surface. The sensitivity of measuring mass gain in a liquid is approximately 1 ng/cm^2^. If the adsorbed mass is evenly distributed in a thin form and is not characterized by high dissipation, the change in the resonance frequency (Δ*f*) is proportional to the adsorbed mass in accordance with the Sauerbrey equation. In the case of viscoelastic layers, the Voight model is used. The QCM-D technique also allows monitoring energy dissipation (Δ*D*), the value of which is correlated with the viscoelastic properties of the adsorbed layer. In the case of layers characterized by a low energy dissipation value Δ*D* < 1 × 10^–6^, we are dealing with the so-called stiff layers that are characterized by low flexibility.

**Figure 8:**
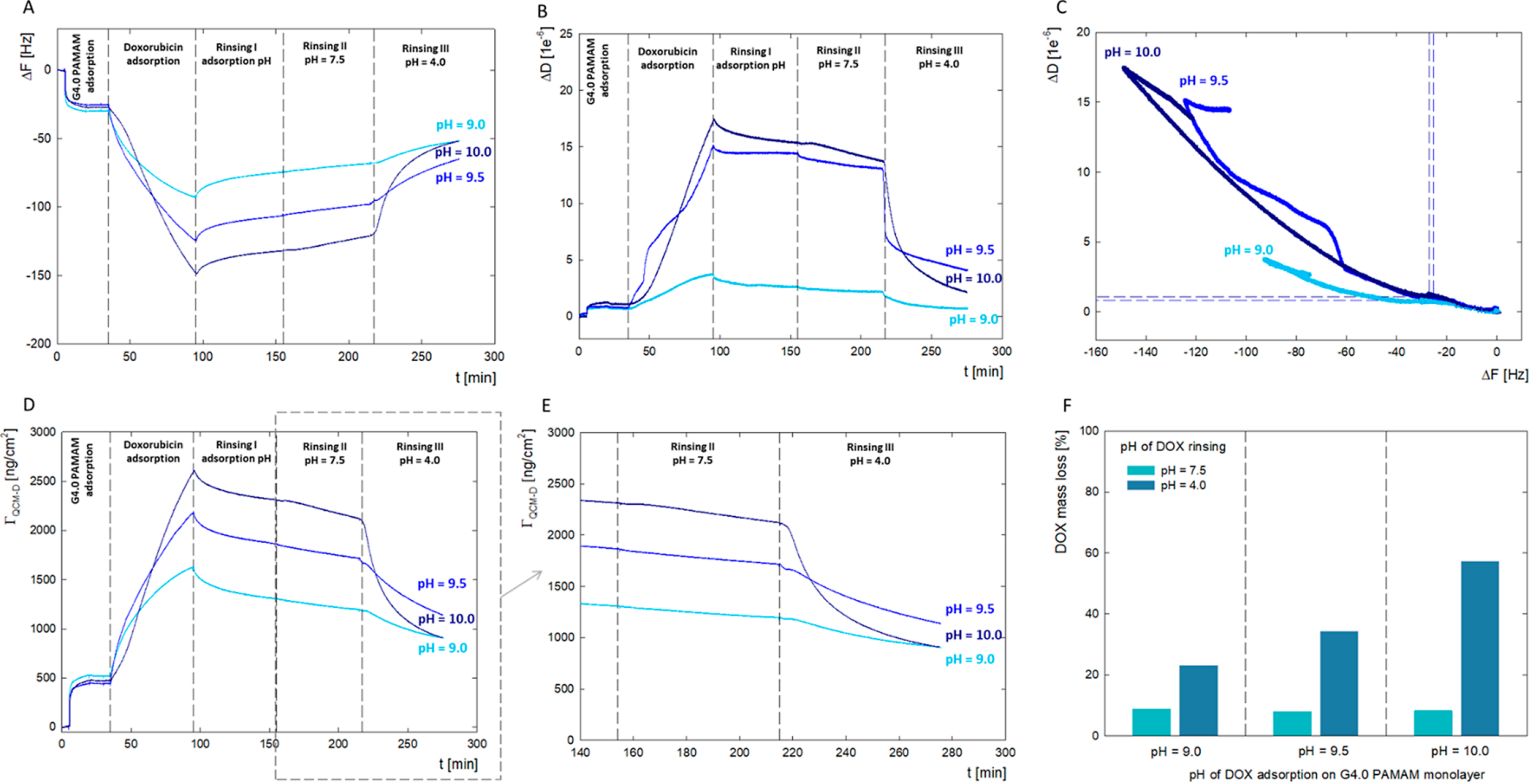
QCM-D results for G4.0-DOX bilayers formation at a pH range of DOX solution for ratio PAMAM/DOX 1:9 at pH 9.0, 9.5, and 10.0. (A) Time dependence of the resonance frequency (Δ*F*) of the QCM-D sensor’s vibrations due to DOX adsorption on dendrimer layer for different pH; (B) time dependence of dissipation changes after DOX adsorbed of the dendrimer layer for different pH; (C) Δ*D* as a function of Δ*F* of the QCM-D sensor as a result of DOX adsorption on the layer of the dendrimer; (D) time dependence of the DOX mass adsorbed on the dendrimer layer for different pH values; (E) time dependence of the DOX mass loss from the dendrimer layer due to changes in the pH of the water solution to pH = 7.5 (rinsing II) and pH = 4.0 (rinsing III); and (F) percentage mass loss of DOX as a function of adsorption conditions (pH = 9.0, 9.5, and 10.0) and rinse pH (pH = 7.5 and 4.0).

As part of the research, the adsorption efficiency of the doxorubicin molecule onto the surface of the dendrimer monolayer was monitored. The dendrimer layer on the sensor surface was adsorbed at pH 10.0 to obtain a high surface coverage. The conditions for obtaining a stable irreversible dendrimer layer were selected based on previous studies.^36^ At pH 10.0, G4.0 PAMAM dendrimers have an isoelectric point, thus forming a compact layer on the sensor surface. The measurements began by washing the sensor with an electrolyte solution of a given pH and ionic strength, and then, after establishing a baseline, changes in the resonance frequency and energy dissipation resulting from the adsorption of dendrimers and doxorubicin were monitored. Dendrimer molecules were adsorbed for 15 min, monitoring the decrease in Δ*f* and the increase in Δ*D* until a plateau was reached. The solvent solution was again passed through the QCM-D cell in the next step. After the base layer based on dendrimers was obtained, a doxorubicin solution with a given pH was introduced into the system. All obtained results are presented for the seventh overtone (*n* = 7) (Figure 8). The adsorbed mass (Γ_QCM-D_) of the layer was calculated by using the QTools software. Doxorubicin interaction with the surface of dendrimers was monitored in the pH range of 7.5 to 10.0. For pH 7.5 and 8.5, no doxorubicin adsorption on the dendrimer layer’s surface was observed. Only for pH > 9.0 is there a change in the sensor’s resonance frequency visible after adding doxorubicin to the system. An increase in pH > 9.0 causes an intense increase in the adsorption of drug molecules.^42^

Under these conditions, large changes in the energy dissipation of the system are simultaneously observed. For the dendrimer layer itself, the dissipation is at the level of Δ*D* = 1 × 10^–6^, typical for stiff layers. Adsorption of doxorubicin at pH 9.5 and 10.0 causes the dissipation to increase to the level of 17 × 10^–6^. The increase in the level of dissipation due to doxorubicin adsorption is not immediate. This increase is observed approximately 15 min after the DOX adsorption.

Based on the surface loading level and the knowledge of the dendrimer molecular weight, it is possible to calculate the number of dendrimer molecules on the interface. The ratio (*N*_DOX_*N*_PAMAM_^–1^) of DOX molecules to the number of dendrimer molecules on the surface is an essential factor describing DOX loading. Figure 8 shows the dependence of the DOX loading ratio on pH. The analysis revealed that the number of doxorubicin molecules per dendrimer increased with increasing pH.

Changes in Δ*D* based on Δ*f* are summarized in Figure 8C. This compares alterations in the layer’s properties with respect to pH at which doxorubicin was adsorbed on the surface of the dendrimers. While the dissipation levels are similar at pH 9.5 and 10.0, the adsorbed mass in these two cases differs significantly. In the curves presented, two adsorption stages can be distinguished, representing the slow and fast phases of the process, which are characterized by a gradient.^50^ In the initial stage of doxorubicin adsorption, the values change slightly. In the case of the fast phase, the slope of the curves increases due to the intensified interaction between the drug and dendrimer molecules. The obtained results indicate a two-step interaction of doxorubicin with the dendrimer. In the first stage, the drug associates on the surface of the dendrimer structure, which, after exceeding a critical concentration, is incorporated into the system and results in an increase in the dissipation of the adsorption layer due to an increase in the hydration of the system. It should be remembered that in the dendrimer structure, there are two different drug localizations on the structure’s surface and inside the system. When considering the binding of DOX through noncovalent interactions to the polymer structure, not only electrostatic interactions but also hydrophobic interactions, π–π stacking, or hydrogen bonds should be taken into account.

Figure 8E shows the process of DOX washout from the surface of a G4.0 PAMAM modified sensor due to a change in the measurement conditions. Washing off with water at pH = 7.5 does not significantly affect the desorption kinetics and slope of the DOX release curve from the carrier surface. This indicates that under physiological conditions, about 8% of the drug mass will be slowly released from the dendrimer surface within an hour (Figure 8F). Significant changes are observed when water flows under acidic conditions (pH = 4.0), resulting in a significant wash-off of 23%–57% of the DOX mass adsorbed on the G4.0 PAMAM surface. Desorption of doxorubicin from the dendrimer structure monitored via QCM-D confirmed the pH-dependent mechanism of drug release. At pH = 4.0, the PAMAM dendrimer swells on the surface of the sensor, and its internal spaces become accessible to the solvent.^36^ In addition, there are changes in the ionization of both the drug molecule and the carrier, resulting in a faster release of doxorubicin from the PAMAM structure.

### Molecular Aspects of G4.0 PAMAM-DOX Complex Formation Using MD

To analyze the interaction of DOX molecules with PAMAM dendrimers under basic and neutral pH conditions, two types of G4.0 PAMAM dendrimers (Figure 1a) at different protonation levels were constructed. Based on acid–base titration experiments^41,43^ conducted at basic pH, all amine groups in the dendrimer were considered unprotonated, while at neutral pH, the primary amines were considered protonated (Figure 1b). We use the abbreviations G4N and G4P to denote the nonprotonated and protonated states, respectively, when referring to high and neutral pH levels. Similar to the PAMAM dendrimer, a DOX molecule can also transform into a series of protonated/deprotonated states. According to the p*K*_a_ of DOX at a high pH (>10), DOX exists in an ionized form due to the deprotonation of the –OH group of the anthracycline moiety (DOX(−), Figure 1c), while at a neutral pH (∼7), DOX is present in a cationic state, thanks to the protonation of the –NH_3_^+^ group [DOX(+), Figure 1d].^43^ During experimental conditions with a pH of around 10, we can assume fully unprotonated amine groups of the dendrimer, with approximately half of the DOX molecules in a neutral state and the second half in an ionized state, DOX(−). Because the negatively charged DOX represents a more challenging case regarding adsorption stability compared with the neutral form, we focused on this more complex scenario in the MD simulations.

**Figure 9:**
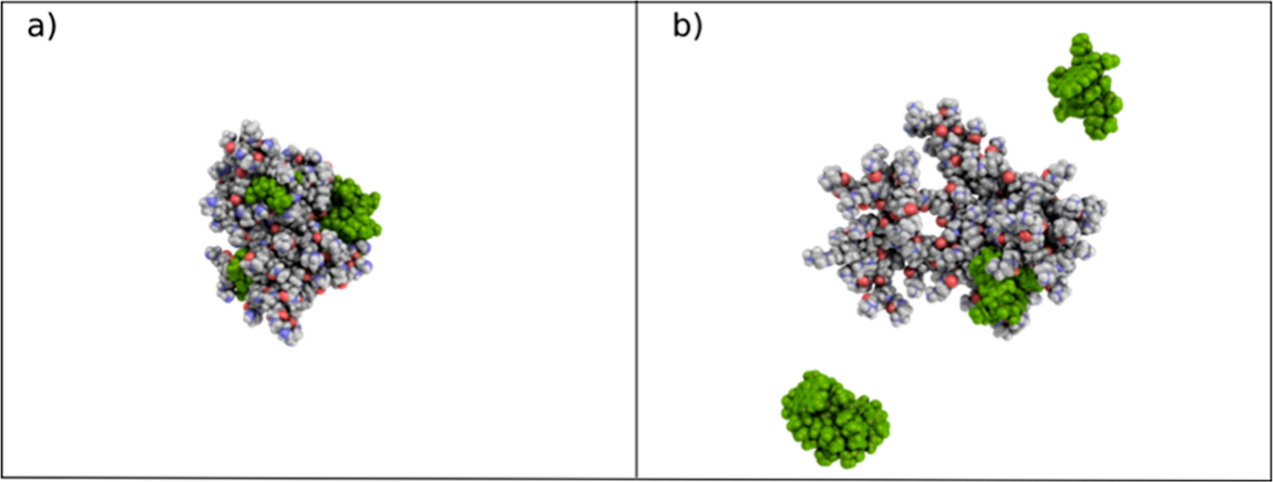
Instantaneous snapshots of (a) G4NP-DOX(−) and (b) G4P-DOX(+) complexes at the end of the simulation runs.

Figure 9 depicts the final configurations of G4N-DOX(−) and G4P-DOX(+) complexes at the end of the MD simulations. In both cases, the ratio of dendrimer to DOX is 1:10, i.e., 10 DOX molecules per a single dendrimer molecule. It is evident from the figure that, under basic pH conditions, all DOX(−) molecules are attracted to the G4NP dendrimer, which would also be the case for neutral DOX molecules (due to the absence of electrostatic repulsion between DOX molecules). In contrast, at a neutral pH, only two DOX(+) molecules interact with the G4P dendrimer, while the remainder of the drug molecules can be found in the bulk water. Additionally, from Figure 9, it is apparent that the G4N dendrimer facilitates the interaction of DOX(−) with both its inner branches and its surface groups. Conversely, the G4.0PAMAM dendrimer allows only DOX(+) interactions with its inner components. This difference can be attributed to the repulsive electrostatic interactions between the protonated primary surface amines of the dendrimer and the –NH_3_^+^ group in DOX. Consequently, DOX(+) adsorption on the G4.0 PAMAM dendrimer can be considered unstable.

**Figure 10:**
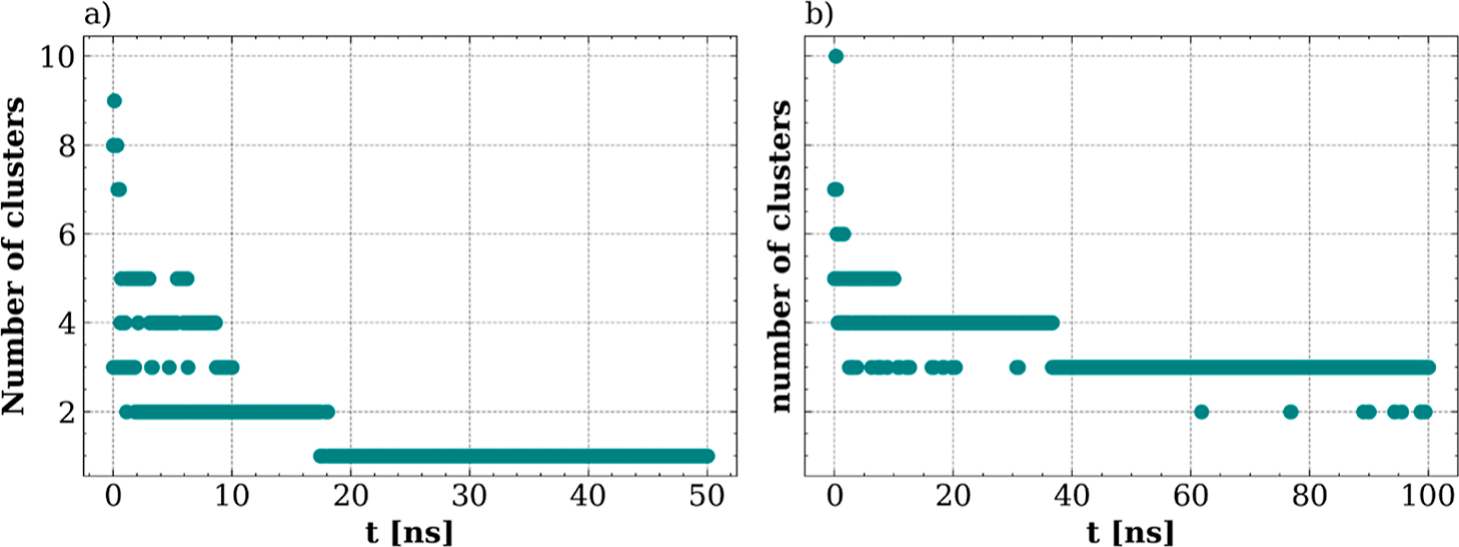
Number of molecular clusters formed in (A) G4N-DOX(−) and (B) G4P-DOX(+) systems during the MD simulations.

To better characterize the dynamic process of DOX adsorption on the dendrimers and the stability of the complexes, we calculated the number of molecular clusters during the MD simulation run. As depicted in Figure 10a, at basic pH, after simulation for 30 ns, the drug molecule and G4N dendrimer form a persistent single molecular cluster. At neutral pH (Figure 10b), the DOX(+) molecules and the G4P dendrimer form three distinct clusters. As observed in Figure 9, these clusters correspond to one cluster of the G4P dendrimer with attached DOX(+) molecules and two DOX clusters located in bulk water. It is also noteworthy that occasionally, the number of clusters drops to two.

Visual inspection of the trajectory revealed that this was related to the anchoring of one of the DOX clusters to the G4P dendrimer. This suggests that at neutral pH, the DOX(+) molecules undergo a dynamic adsorption/desorption process from the G4P dendrimer surface, while at basic pH, the drug molecules and G4NP dendrimer form a stable complex.

**Figure 11:**
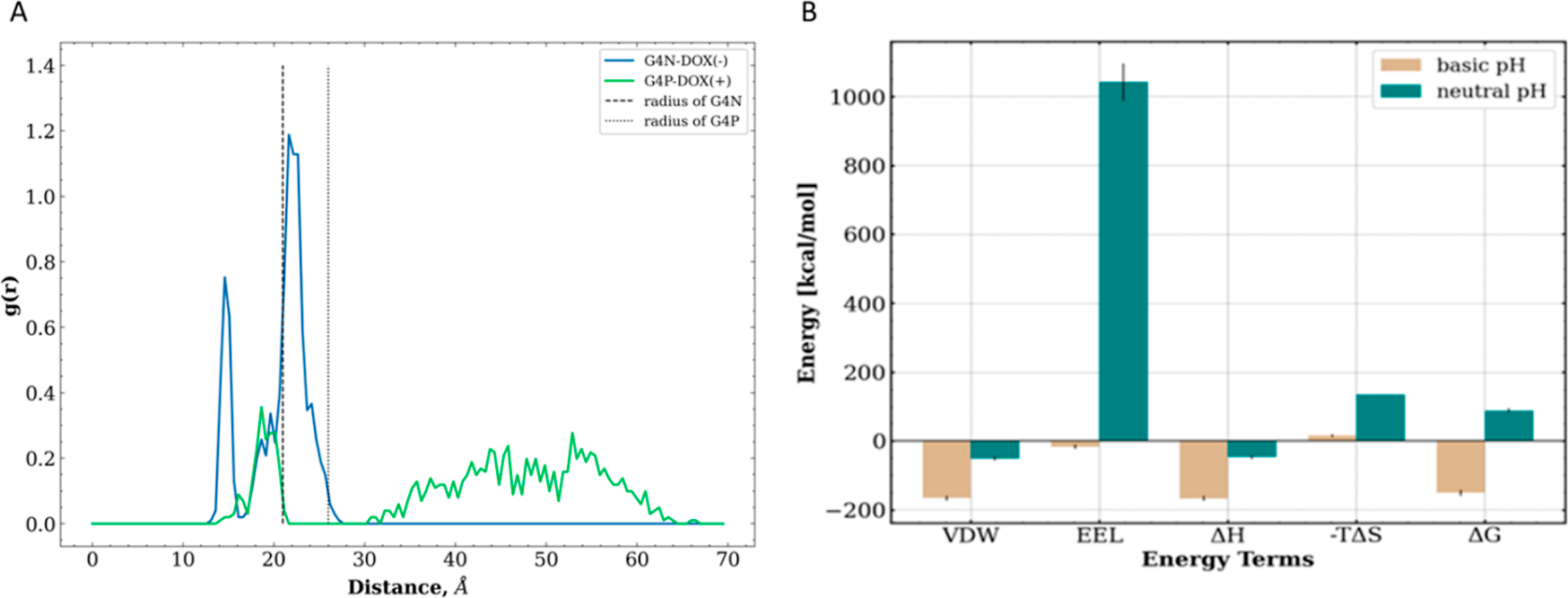
(A) Radial distribution profiles were determined for the distances between the dendrimer and DOX molecules. The dashed vertical lines indicate the radii of the dendrimers; (B) binding free energy values Δ*G* and their decomposition terms for both systems [VDW—van der Waals (VDW) interaction energy, EEL—electrostatic interaction energy, Δ*H*—enthalpic contributions, and *T*Δ*S*—entropy contribution].

To characterize the average positions of drug molecules within complexes, we calculated the radial distribution profiles of the center of mass of DOX molecules with respect to the center of mass of the dendrimers. These profiles are shown in Figure 11. We can see that the radial distribution profile of DOX(−) molecules in the G4N dendrimer exhibits two peaks: the first (smaller) at a distance of approximately 14 Å and the second (higher) at a distance of around 22 Å from the core of the dendrimer. The radius (*r*) of the G4N dendrimer is close to 21 Å, i.e., √(5/3) *R*_g_, where *R*_g_ represents the average radius of gyration for G4N. This indicates that most of the drug molecules are located on the surface as well as in the inner region of the G4N dendrimer. In the case of the G4P dendrimer, the distribution profile for DOX(+) molecules shows a small maximum at a distance of about 18 Å, followed by a broader distribution zone extending from 30 up to 60 Å, which is much larger than the radius of the G4P dendrimer (i.e., approximately 26 Å). This suggests that at neutral pH, DOX drug molecules are primarily distributed in water but can also be bound to the internal pocket of the G4P dendrimer.

To quantify the adsorption affinity of drug molecules to dendrimers, we calculated the binding free energy Δ*G* between DOX and dendrimers in both systems. From Figure 11B, we observe that the G4N-DOX(−) complex exhibits a favorable (negative) value of binding free energy. In contrast, the positive Δ*G* value of the G4P-DOX(+) complex suggests that the self-assembly process of the G4P-DOX(+) nanosystem is thermodynamically unfavorable. Decomposing the Δ*G* value into its enthalpic (Δ*H*) and entropic (−*T*Δ*S*) components reveals that the DOX-dendrimer interaction is predominantly enthalpic in nature. Therefore, the less efficient nanoassembly of the G4P-DOX(+) complex can be rationalized by considering the high number of unfavorable electrostatic interactions (EEL), leading to a decrease in Δ*H*. On the other hand, the favorable VDW energy values reported for both complexes indicate that the nonpolar interactions are the driving force behind complex stabilization.

The computational data qualitatively support experimental observations regarding the adsorption of DOX on G4 PAMAM dendrimers. Furthermore, a deeper understanding of the structure of G4-DOX complexes was obtained, leading to the conclusion that DOX primarily binds to the surface of the dendrimers but DOX(−) can also penetrate the inner region of the G4N dendrimers. DOX(+), on the other hand, binds weakly, and only a small number of drug molecules interact directly within the inner skeleton of the dendrimer. This preference is likely due to its uncharged state, unlike the terminal parts of the dendrimer, which carry positively charged amine groups. Quantitatively, it is confirmed that stable complexes formed under basic conditions will lose stability upon pH reduction. This phenomenon can be exploited in the construction of drug-releasing platforms.

## Conclusions

To summarize, our studies illustrate that doxorubicin in the physiological environment occurs in the protonated form, and at a higher pH, it takes on the deprotonated form. These are ideal conditions for forming a complex between positively charged G4.0 PAMAM dendrimers and negatively charged doxorubicin. The successive immobilization of DOX to the G4.0 PAMAM structure was monitored by changes in the electrophoretic mobility values of the formed complex and UV–vis, FTIR, and CD spectroscopy. Using the QCM-D method, the influence of pH on the efficiency of the formation of the G4.0 PAMAM-DOX complex under dynamic conditions was tested. Effective adsorption of DOX to the dendrimer layer was observed for pH > 8.5, and the highest for pH = 10.0. Environmental conditions significantly affect the viscoelastic properties of the formed G4.0 PAMAM/DOX bilayer. It should be emphasized that the presence of drug molecules in the dendrimer structure causes a significant increase in the system’s hydration. Under basic conditions, the deprotonated form of the drug predominates, which is immobilized on the surface of the carrier through electrostatic interaction; other tautomeric forms occur under these conditions and may prefer to be located in the hydrophobic interior of the polymer. Computational studies have provided more profound insights into the adsorption mechanism of DOX on the structures of PAMAM dendrimers. The conclusions drawn from these studies fully support experimental observations regarding the stability of the complexes at basic pH and their ability to release the drug under lower pH conditions. The location of the drug in the carrier structure is crucial in the context of the drug release rate from the carrier structure as well as its stability and activity under biological conditions.

## Supporting Information Available

Changes in the potential energy and RMSD values of the simulated systems (PDF)
