# Supplementary material for: Theoretical and Experimental Analyses of the Interfacial Mechanism of Dendrimer–Doxorubicin Complexes Formation
Source: Mol Pharm. 2024 Oct 22;21(11):5892–904. doi: 10.1021/acs.molpharmaceut.4c00941 (PMC11539063; doi:10.1021/acs.molpharmaceut.4c00941)
Supplement: Supplementary file 1 — mp4c00941_si_001.pdf [file mp4c00941_si_001.pdf]

Supplementary Information

A Theoretical and Experimental Analysis of the Interfacial Mechanism of Dendrimer-Doxorubicin Complexes Formation

Barbara Jachimska[1], Magdalena Goncerz[1], Paweł Wolski[1], Callum Meldrum[2], Łukasz Lustyk[1], Tomasz Panczyk[1],*

[1] Jerzy Haber Institute of Catalysis and Surface Chemistry Polish Academy of Sciences, Krakow, Poland

[2] Department of Chemical and Process Engineering, University of Strathclyde; 75 Montrose Street, Glasgow G1 1XJ, UK

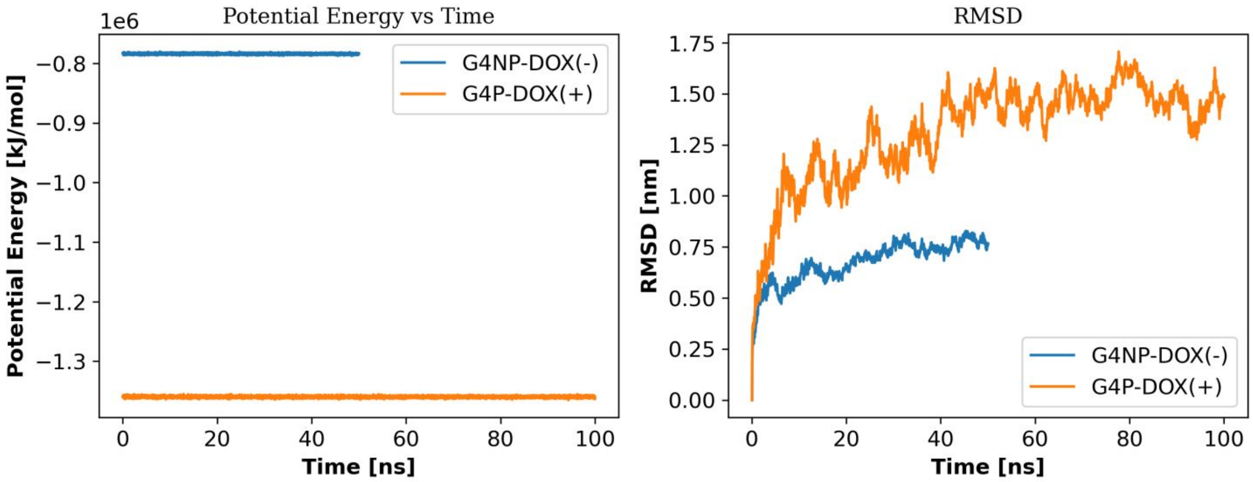

Figure S1. Change in the potential energy (left) and RMSD (right) values as a function of time. The RMSD value was calculated only for the dendrimer using the initial structure as a reference.
